# A Composite Membrane with High Stability and Low Cost Specifically for Iron–Chromium Flow Battery

**DOI:** 10.3390/polym14112245

**Published:** 2022-05-31

**Authors:** Lin Qiao, Shumin Liu, Maolin Fang, Mingjun Yang, Xiangkun Ma

**Affiliations:** Department of Materials Science and Engineering, Dalian Maritime University, Dalian 116026, China; qiaolin2020@dlmu.edu.cn (L.Q.); shumin9801@163.com (S.L.); fangml@dlmu.edu.cn (M.F.); ymsj@dlmu.edu.cn (M.Y.)

**Keywords:** iron–chromium flow battery, pore-filling Daramic membrane, ion selectivity, low cost, high stability

## Abstract

The iron–chromium flow battery (ICFB), the earliest flow battery, shows promise for large-scale energy storage due to its low cost and inherent safety. However, there is no specific membrane designed that meets the special requirements of ICFBs. To match the harsh operation parameters of ICFBs, we designed and fabricated a composite membrane with high mechanical, chemical, and thermal stability. In the design, a commercial porous polyethylene membrane is selected as the framework material, offering high mechanical stability and reducing the cost. Meanwhile, the Nafion resin is filled in the pores of a porous membrane, which inhibits the transfer of redox-active ions and creates the proton channels via hydrophobic/hydrophilic phase separation. As a result, the composite membrane exhibits high conductivity, selectivity, and stability, especially with almost no swelling at high operating temperatures. Thus, an ICFB with the prepared membrane exhibits a coulombic efficiency of 93.29% at the current density of 80 mA cm^−2^ and runs stably for over 300 cycles. This work provides an easy method to fabricate high-performance and low-cost membranes specifically for ICFBs and has the potential to promote the development of ICFBs.

## 1. Introduction

With the blooming development of intermittent renewable energy sources [1], such as wind and solar energy, large-scale energy storage technology is in urgent need to smooth out the load-leveling of a power grid system [2]. For large-scale energy storage applications, high safety, long life, and low cost are required [3]. Aqueous flow batteries are one of the promising candidates [4,5,6] due to their use of non-flammable electrolytes and unique independently tunable power and capacity features. In existing aqueous flow batteries, vanadium flow batteries (VFB) are the most mature ones, however, the high price of vanadium ore constrains their further commercial development [7].

In order to reduce the cost of aqueous flow batteries, increasing attention is focused on low-priced redox-active material [8]. Iron–chromium flow batteries (ICFB) with low-cost electrolytes have again appeared in sights of researchers after their debut in 1974 [9,10,11,12]. Recently, the demonstration projects of MWh-level ICFBs were established in the USA and China [4,13]. However, many challenges limit the market penetration of ICFBs, including low efficiency, serious capacity decay, and poor long-term stability [2,4]. These challenges are mainly attributed to the sluggish redox kinetics of the Cr^2+^/Cr^3+^ couple. Many hard efforts, such as a high operating temperature (65 °C) [14], high electrode compression ratio, and high flow rate [15], are imposed to improve the kinetics. Meanwhile, these operation parameters aggravate the cross-over of redox-active species and bring stricter requirements for the stability of the key materials, in particular the ion conductive membrane.

An ion conductive membrane, as a key component of an ICFB, plays the role of preventing the cross-over of positive and negative active species while conducting the protons to complete the electrical circuits [16]. The properties of a membrane greatly affect the performance of ICFBs, especially under harsh conditions. An ideal membrane in an ICFB is required to possess these features, including low swelling at 65 °C, high thermal and mechanical stability, low cost, and high selectivity and conductivity.

However, a few researchers focus on the specific membrane for ICFBs. Given that the stability of anion exchange membranes is still a serious problem, membranes commonly used in ICFBs are cation exchange membranes learning from VFB [17,18,19] or other batteries [20,21,22,23,24], such as perfluoro sulfonic membranes like the Nafion series [25] or sulfonated polyether ether ketone (SPEEK) membranes [26,27]. Nafion membranes are used widely in other battery systems due to their high chemical stability and high proton conductivity. Nevertheless, in ICFBs, Nafion membranes face serious swelling because of the high operating temperature, leading to low ion selectivity. Meanwhile, the carbon fibers of highly compressed electrodes carry the risk of penetrating the highly swollen Nafion membranes at 65 °C. Besides, the extremely high price significantly raises the cost of the ICFB system. Recently, SPEEK membranes with low cost have been applied in ICFBs. However, SPEEK membranes with a low sulfonation degree show poor proton conductivity. Also, the usage of SPEEK membranes with a high sulfonation degree are limited by serious swelling and poor chemical and mechanical stability. Therefore, it is highly desirable to develop a novel membrane specifically designed to meet the special requirements of ICFBs.

**Scheme 1:**
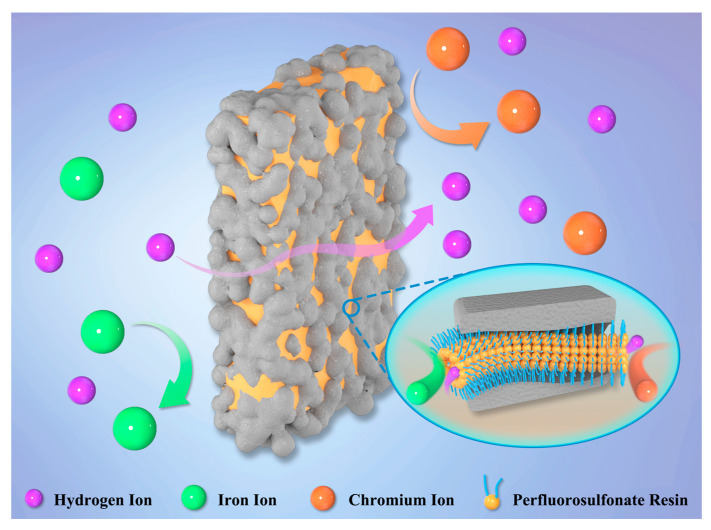
The transport mechanism of the composite membrane for iron–chromium flow battery.

Herein, we designed and prepared a composite membrane with high thermal and mechanical stability for ICFBs via a simple method. In the design, a commercial porous polyethylene membrane (Daramic) with low cost and high mechanical stability is selected as the framework material, while the Nafion resin with high ion conductivity is filled into the pores of the Daramic porous membrane, as shown in Scheme 1. The confinement effect of the porous framework restricts the serious swelling and the obvious hydrophobic/hydrophilic phase separation of the Nafion resin at 65 °C, dramatically improving the ion selectivity and stability. In addition, the composite membrane chooses the cheap Daramic membrane (31.525 $m^−2^) as its main material, reducing the amount of expensive Nafion resin and drastically cutting the cost. The cost of the prepared membrane is less than 50% of the recast Nafion 115 membrane by Nafion dispersions. In the paper, the casting method, the composition, and the amount of cast solution are discussed in detail and optimized. As a result, a composite membrane with high stability and low cost is prepared successfully. An ICFB with the prepared membrane exhibits outstanding performance.

## 2. Experimental Section

### 2.1. Materials

Daramic membranes were obtained from Daramic LLC. Daramic (Shanghai, China) battery membrane Co., Ltd. Nafion dispersions (D-2020) and Nafion 115 membrane were purchased from the Chemours company from Shanghai of China. N,N-Dimethylacetamide (DMAc, Zhongtian Fine Chemicals Co., Ltd., >99.5%, Shenyang, China), Isopropanol (IPA, Damao, >99.7%), Iron (II) chloride tetrahydrate (FeCl_2_·4H_2_O, Ourchem, 99%), Chromium chloride hexahydrate (CrCl_3_·6H_2_O, Damao, >99%), Iron trichloride hexahydrate (FeCl_3_·6H_2_O, Damao, >99%), Silver nitrate (AgNO_3_, Kermel, >99.8%), Aluminum chloride (AlCl_3_, Aladdin, 99%), Hydrochloric acid (HCl, Shenyang Paier Fine Chemical Co., Ltd., >36%, Shenyang, China), and Sulfuric acid (H_2_SO_4_, Shenyang Paier Fine Chemical Co., Ltd., >95%, Shenyang, China), were used without further purification. The carbon felt was received from Liaoyang Jingu Co., Ltd. from Liaoyang of China.

### 2.2. Membrane Preparation

First, the 2 mL of 5 wt% Nafion dispersion were cast on Daramic membrane with an area of 8 cm × 8 cm by the drop-casting method, and then Daramic membrane with Nafion dispersion was dried at 60 °C. The process was repeated until the amount of Nafion was enough. The 5 wt% Nafion dispersion was obtained by diluting a 20 wt% Nafion dispersion (D-2020) with deionized water, IPA, or DMAc. Finally, the prepared membranes were pretreated by sequential immersion in 3% H_2_O_2_, 0.5 M H_2_SO_4_, and deionized water for 1 h at 80 °C.

### 2.3. Chemical Structure

The chemical structure of the composite membrane was characterized by Fourier transform infrared spectroscopy (JASCO FTIR 4100).

### 2.4. Membrane Morphology

The morphology of the composite membrane was recorded by scanning electron microscopy (SEM), transmission electron microscopy (TEM), and atomic force microscopy (AFM). The SEM samples were coated with gold and the cross-section ones were fractured in liquid nitrogen. The TEM samples were prepared by the following procedures: first, immerse the composite membranes in 0.05 M AgNO_3_ solution to replace H^+^ with Ag^+^, then the composite membranes were fixed in epoxy resin and cut into thin slice samples. The AFM experiments were carried out with NT-MDT in the tapping mode.

### 2.5. Water Uptake

The water uptake (WU) of the membrane was calculated according to the weight change of dry and wet membrane. Firstly, the membrane was soaked in deionized water for 24 h to make it fully infiltrated. Then, we wiped up the surface moisture with filter paper and quickly weighed it as W_wet_. Finally, the membrane was dried at 60 °C for 24 h and weighed as W_dry_. The WU was calculated with Formula (1):

(1) $$\mathrm{WU}\;\left(\%\right)=\frac{W_{\mathrm{wet}}-W_{\mathrm{dry}}}{W_{\mathrm{dry}}}\times 100\%$$

### 2.6. Contact Angle

The static contact angle on the surface of the membranes was measured by the contact angle tester (Shanghai zhongchen digital technic apparatus Co., Ltd., Shanghai, China).

### 2.7. Iron Ion Permeability

The iron ion permeability was evaluated by a diffusion cell with 3 × 3 cm^2^ composite membranes. The left chamber was filled with 80 mL 1 M FeCl_3_ in 3 M HCl solution and the right chamber was filled with 80 mL 1 M AlCl_3_ in 3 M HCl solution to balance the osmotic pressure. The solution in both chambers was vigorously stirred to reduce the concentration polarization. The concentration of Fe^3+^ in the right chamber was detected via a UV-vis spectrometer (JASCO, FT-IR 4100, Tokyo, Japan) at a regular time interval.

### 2.8. Area Resistance

The area resistance was measured via a conductive cell filled with 0.5 M H_2_SO_4_ solution. First, we measured the resistance value of the blank cell without the membrane as R_1_. Second, we put the membrane with an effective area of 1 × 1 cm^2^ into the conductive cell and measured the resistance value of the cell as R_2_. Finally, resistance R of the measured membrane was calculated by the following Formula (2):

(2) $$R=R_{2}-R_{1}$$

### 2.9. Battery Performance

The battery was assembled by a method similar to the one reported in our previous work [27]. Briefly, the ICFB was assembled by sandwiching membrane between two carbon felt electrodes, two graphite polar plates, and two endplates. The effective area of the membranes is 6 × 6 cm^2^ and the carbon felt electrode with a thickness of 6 mm was compressed to 3 mm. A composition of 1 M CrCl_3_ and 1 M FeCl_2_ in 3 M HCl solution was used as the electrolyte and driven by the peristaltic pump, where 50 mL of electrolyte flows through the electrode with a flow rate of 50–60 mL min^−1^. The carbon felt electrodes were activated by immersing the concentrated sulfuric acid for 24 h before use. The galvanostatic charge–discharge tests of the ICFB were conducted by a battery testing system (LAND, CT2001A) at 65 °C. The current densities were set in the range of 40 to 80 mA cm^−2^. The charge and discharge cut-off voltage were set at 1.2 V and 0.8 V, respectively. For the open-circuit voltage (OCV) curve test, the ICFB was charged to 50% SOC and stopped when the OCV was lower than 0.2 V. For the polarization test, the ICFB was charged to 80% state-of-charge (SOC) and 50% SOC, which was assembled by a membrane with an effective area of 3 × 3 cm^2^. After, the ICFB was discharged at different current densities and the respective voltages were recorded. The coulombic efficiency (CE), voltage efficiency (VE), and energy efficiency (EE) can be obtained from the following Formulas (3)–(5):

(3) $$\mathrm{CE}\;=\frac{\mathrm{Discharge}\;\mathrm{Capacity}}{\mathrm{Charge}\;\mathrm{Capacity}}\times 100\%$$

(4) $$\mathrm{EE}\;=\frac{\mathrm{Discharge}\;\mathrm{Energy}}{\mathrm{Charge}\;\mathrm{Energy}}\times 100\%$$

(5) $$\mathrm{VE}\;=\frac{\mathrm{EE}}{\mathrm{CE}}\times 100\%$$

### 2.10. Stability Test

The thermal stability of membranes was evaluated by thermogravimetric (NETZSCH STA 449 F5/F3 Jupiter) and swelling tests. The swelling ratio (SR) of the membrane was calculated according to the size change of the dry and wet membrane. Firstly, the membrane was soaked in deionized water for 24 h to make it fully infiltrated. Then, we wiped up the surface moisture with filter paper and quickly measured the length as L_wet_. Finally, the membrane was dried for 24 h and its length was measured as L_dry_. The SR was calculated with Formula (6):

(6) $$\mathrm{SR}\;\left(\%\right)=\frac{L_{\mathrm{wet}}-L_{\mathrm{dry}}}{L_{\mathrm{dry}}}\times 100\%$$

The mechanical stability of the membranes was evaluated by the AG-2004 (Shimadzu) universal strength tester, where the effective area of the membranes is 1 cm × 5 cm. The puncture resistance was investigated by an electromechanical universal testing machine (Zwick Z2.5), where the effective area of the membranes is a circle with a radius of 10 cm.

The DSC measurement was carried out by QS-2000 differential scanning calorimeter at a heating rate of 10 °C min^−1^ and a temperature range from 30 °C to 200 °C.

The chemical stability of the membranes was tested by the change of mass before and after immersing in Fenton’s reagent (consisting of 3% H_2_O_2_ and 2 ppm FeSO_4_) at 80 °C for 7 h.

## 3. Results

### 3.1. The Fabrication of the Composite Membranes

To construct the design, the composite membranes were prepared by a simple drop-coating method. The coating way and the concentration of the cast solution have major impacts on the surface uniformity of the composite membranes. The composite membranes prepared by the cast solution with a high concentration or one-step coating way were seriously inhomogeneous (Figure S1) due to the high viscosity. Therefore, the 5 wt% cast solution was selected and the step-by-step coating way was proposed, wherein the prepared composite membrane showed a uniform surface (Figure S2).

The 5 wt% cast solution was obtained by diluting 20 wt% Nafion dispersion (D-2020). The performance of the composite membranes is closely related to the sort of diluting solvent. The solvent of 20 wt% Nafion dispersion is mainly water and isopropanol (IPA). Also, N,N-Dimethylacetamide (DMAc) is a common solvent for cast solutions. Thus, deionized water, IPA, and DMAc were chosen as diluting solvents. The composite membranes were prepared and referred to as the D-H_2_O, D-IPA, and D-DMAc membrane, respectively, where the 6 mL cast solution was coated 3 times.

**Figure 1:**
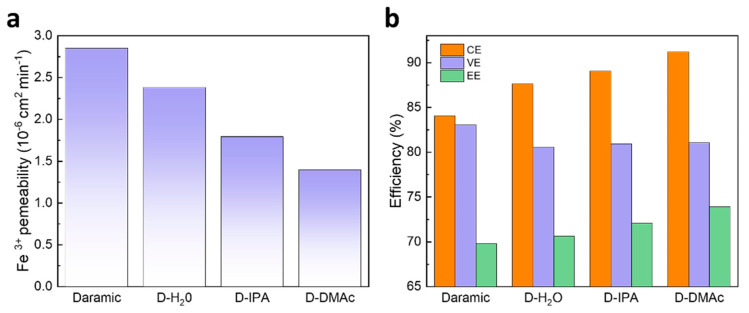
(**a**) Iron ion permeability of Daramic, D-H_2_O, D-IPA, and D-DMAc membranes; (**b**) The performance of ICFBs with Daramic, D-H_2_O, D-IPA, and D-DMAc membranes at the current density of 80 mA cm^−2^.

The ion selectivity of the membrane has a great effect on the capacity retention and coulombic efficiency (CE) of an ICFB. Firstly, the ion selectivity of the Daramic, D-H_2_O, D-IPA, and D-DMAc membranes were evaluated by Fe^3+^ permeability. As shown in Figure 1a, Daramic membranes exhibit serious Fe^3+^ permeance due to their large pores. Compared with Daramic membranes, the composite membranes showed lower permeability due to the coating of Nafion resin, which means the ion selectivity was improved by the drop-coating method. In addition, the obvious different permeability was observed for the D-H_2_O, D-IPA, and D-DMAc membranes, and the permeability followed the sequence: D-H_2_O membrane > D-IPA membrane >D-DMAc membrane.

The ICFBs were assembled and detected to evaluate the effects of the Nafion resin coating. The performance of ICFBs with Daramic, D-H_2_O, D-IPA, and D-DMAc membranes at the current density of 80 mA cm^−2^ are shown in Figure 1b. In accordance with the results of Fe^3+^ permeability, ICFBs with the composite membranes exhibit a higher CE than those with a Daramic membrane, and an ICFB with a D-DMAc membrane achieves the highest CE of 91.18%. It’s worth noting that the voltage efficiency (VE) of ICFBs assembled with the composite membranes has little change compared with that of the Daramic membrane. The results above indicate that the composition of the cast solution greatly affects the ion selectivity and has almost no influence on ion conductivity.

**Figure 2:**
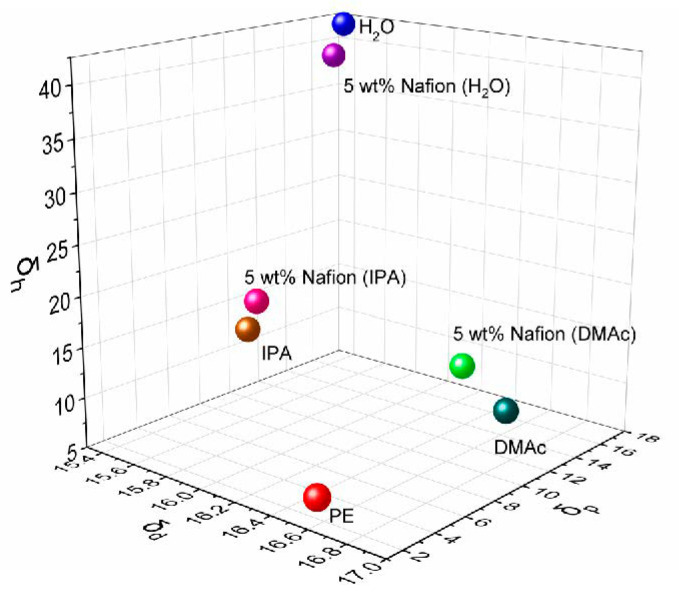
Solubility parameter of the different solution.

The obvious difference of ion selectivity for the D-H_2_O, D-IPA, and D-DMAc membranes is attributed to the different affinities between the solution and Daramic membrane. The solvent composition of the 5 wt% cast solution was listed in Table 1. The solubility parameters (δ, δ_d_, δ_p_, and δ_h_) of the mixed solvents were calculated based on the solvent composition (Table 1 and Figure 2). The main polymer of Daramic membrane is polyethylene (PE), with a solubility parameter δ of 17.8 and experimentally-determined solubility sphere radius (Ro) of 7.9 [28]. The solubility parameter “distance” (Ra) between the mixed solvent and polymer PE was calculated and shown in Table 1.

In general, when the Ra value of the mixed solvents is less than Ro of the polymer, this solvent is referred to as a “good” solvent and will dissolve or swell the polymer. That is, the difference between Ra and Ro determines their affinity. As shown in Table 1, the difference (Ra-Ro) from the mixed solvent of D-DMAc is the smallest, which is consistent with the solubility parameter difference (∆δ). Meanwhile, the solvent composition of D-DMAc is the closest to the “solubility circle” (Figure 2) of PE, while that of D-H_2_O is far away from the circle. Namely, the affinity between the mixed solvents and PE is in the order of D-DMAc-PE > D-IPA-PE > D-H_2_O-PE.

According to the analysis of the solubility parameter, the Daramic membrane with PE as the main polymer prefers swelling in the mixed solvent of 5 wt% Nafion (DMAc) to that of 5 wt% Nafion (H_2_O) or 5 wt% Nafion (IPA). For this reason, in the drop-coating process, it is easier for Nafion resin in the mixed solvent of 5 wt% Nafion (DMAc) to get into the swelling pores of the Daramic membrane. For D-DMAc membrane, the more Nafion resin in the pores erects more barriers against the ion crossover, helping D-DMAc membrane achieve a higher ion selectivity. Simultaneously, the massive Nafion resin in the pores would form the ion channels via hydrophobic/hydrophilic phase separation, ensuring the transfer of protons. Therefore, the composite membrane using 5 wt% Nafion (DMAc) as a diluting solvent exhibits the delighted ICFB performance.

### 3.2. The Characterizations of the Composite Membranes

**Figure 3:**
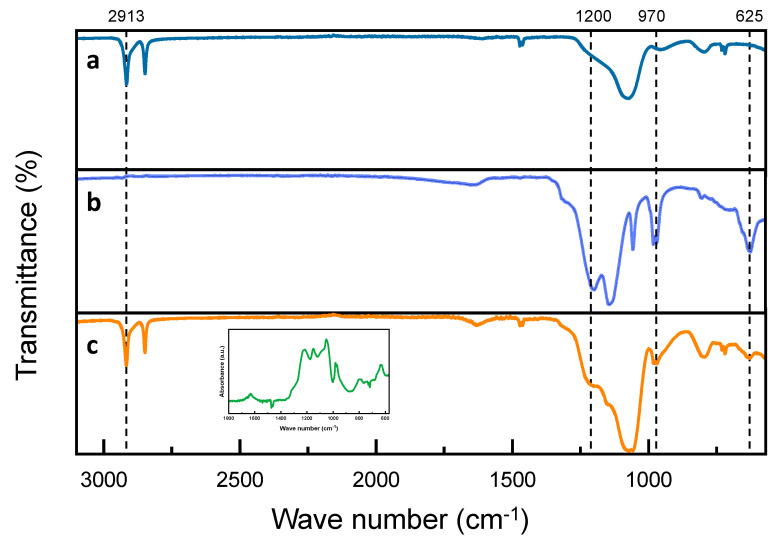
FT-IR of (**a**) Daramic; (**b**) Nafion; and (**c**) D-DMAc membranes—inset in (**c**): the difference spectrum of D-DMAc membrane subtracting the Daramic curves.

Subsequently, we characterized the chemical structure of the D-DMAc membrane. For comparative analysis, Fourier transforms infrared (FTIR) spectroscopy of Daramic and Nafion 115 membranes were also performed. In Figure 3a, for Daramic membrane, the bands at 2913 and 2848 cm^−1^ correspond to the asymmetrical and symmetric stretching vibration of -CH_2_ in PE [29], and the band at 1073 cm^−1^ corresponds to the stretching vibration of Si-O-Si [30]. For the D-DMAc membrane, compared with the Daramic membrane, new peaks at 1200, 1143, 982, 970, and 625 cm^−1^ appear, which is attributed to the stretching vibration of C-F and C-O-C in the Nafion resin [31]—and is also verified in the inset of Figure 3c. This demonstrates that Nafion resin has been successfully introduced into the Daramic membrane.

**Figure 4:**
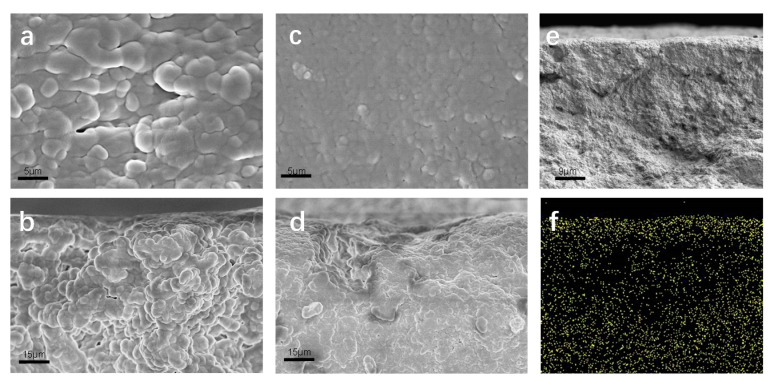
The (**a**) surface and (**b**) cross-sectional SEM images of Daramic; the (**c**) surface and (**d**,**e**) cross-sectional SEM images of the D-DMAc membrane; (**f**) EDS mapping results of the D-DMAc membrane.

To further clarify the distribution of Nafion resin, the surface and cross-section morphologies of the D-DMAc and Daramic membranes were recorded by scanning electron microscopy (SEM). Obviously, in the SEM images (Figure 4), the Daramic membrane exhibits a porous structure with nano-size. The surface and cross-section morphologies of D-DMAc become dense and smooth and few pores were observed after the coating of Nafion resin.

Combining the EDS mapping (Figure 4f) results, the Nafion resin (characteristic fluorine element) is observed on the surface and cross-section of D-DMAc. These above results demonstrate that Nafion resin successfully filled in the pores of the Daramic membrane via the simple drop-coating method.

**Figure 5:**
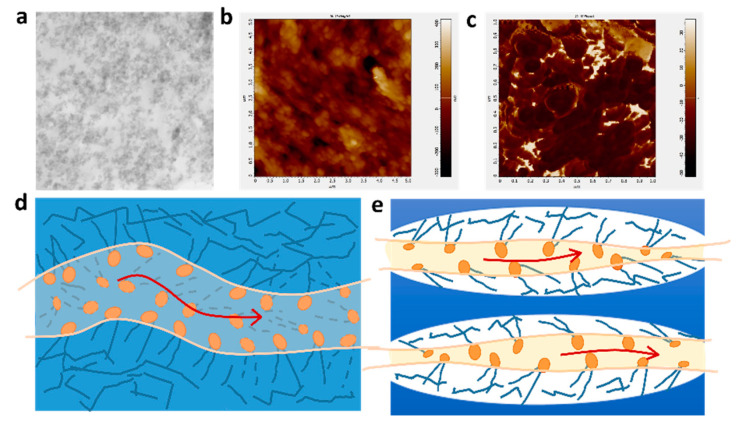
(**a**) Surface TEM images, (**b**) AFM topography images, (**c**) AFM phase images of D-DMAc-8 membrane; The schematic diagram of hydrophilic ion channels in (**d**) Nafion and (**e**) D-DMAc membranes.

The transmission electron microscopy (TEM) images are shown in Figure 5a. The D-DMAc membrane was dyed with AgNO_3_ before TEM tests to replace H^+^ in Nafion resin with Ag^+^. The regions with dark dots correspond to the distribution of silver ions (Ag^+^), that is, the sulfonated groups in Nafion resin. Hydrophobic segments of the polymerization backbone are bright regions, while clusters of hydrophilic ions of the polymer are dark. We can clearly see that the hydrophilic sulfonic acid groups are evenly dispersed in the image. Meanwhile, in the atomic force microscopy (AFM) phase images, we can also see the bright and dark regions (Figure 5b,c)—where the dark and bright regions correspond to the soft hydrophilic and hard hydrophobic domains, respectively. Thus, the Nafion resin in the pores would form the proton channels via hydrophobic/hydrophilic phase separation, ensuring the transfer of protons. In addition, the confinement effect of the porous framework restricts the obvious hydrophobic/hydrophilic phase separation of Nafion resin (Figure 5d,e).

### 3.3. The Optimization of Nafion Resin Amount

**Figure 6:**
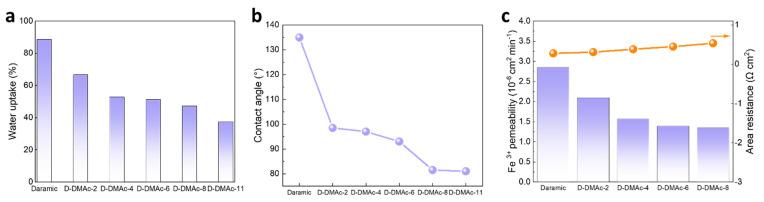
(**a**) Water uptake (WU); (**b**) Contact angle; and (**c**) Iron ion permeability and area resistance of D-DMAc-x membranes.

Further, the amount of Nafion resin was optimized by regulating the coating times. The prepared membranes are named as D-DMAc-x membrane, where x represents the volume of 5 wt% Nafion solution. To cover the membrane evenly, the 3 mL volume was used as the fifth coating. The physicochemical properties of D-DMAc-x membranes, including WU and contact angle, are tested. As shown in Figure 6a, the Daramic membrane had high WU due to its large pore size. Also, WU decreases with an increasing volume of Nafion solution, exhibiting that the Nafion resin successfully filled its pores (Figure 6a). The results of the contact angle are shown in Figure 6b. With the addition of Nafion resin (2–8 mL), the contact angle decreases sharply from 135° to 81.5° (Figure S3), that is, the hydrophobic membranes change to the hydrophilic type by coating the Nafion resin. The improved hydrophilicity helps protons pass rapidly through D-DMAc-x membranes via the vehicle mechanism. Also, the contact angle of D-DMAc-11 is comparable to that of D-DMAc-8, meaning the pores in the Daramic membrane have been filled in completely by 8 mL Nafion solution. Therefore, the D-DMAc-8 membrane is expected to show the best comprehensive performance.

To investigate the ion transport properties of D-DMAc-x membranes, we measured the Fe^3+^ permeance and area resistance. As shown in Figure 6c, the permeation of Fe^3+^ is suppressed by the coating of Nafion resin. In addition, as the volume of Nafion solution increases from 2 mL to 8 mL, the Fe^3+^ permeance was significantly reduced. In general, the decreasing ion permeability would lead to worse ion conductivity. The area resistance of D-DMAc-x has a slight rise with the rising amount of Nafion resin. These results indicate that the coating of Nafion resin has little effect on the transport of protons, which is attributed to the high hydrophilicity and high ion conductivity of the Nafion resin.

### 3.4. ICFB Battery Performance

**Figure 7:**
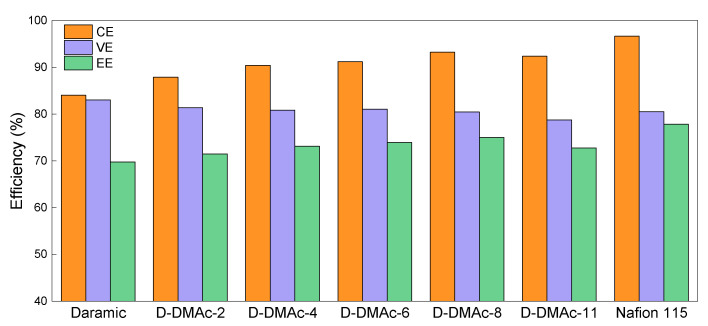
ICFB performance of Daramic, D-DMAc-x (x = 0–11), and Nafion 115 membranes at the current density of 80 mA cm^−2^.

ICFBs with D-DMAc-x (x = 0~11) membranes were assembled to evaluate the effects of the Nafion resin amount. Compared with Daramic membrane, a drastic increase in CE (from 84.05% to 93.29%, at the current density of 80 mA cm^−2^) is achieved by the coating of Nafion resin (Figure 7). Besides, CEs of ICFBs with D-DMAc-x membranes first rise with the rising Nafion resin amount and then reach a stable value, which is in accordance with the Fe^3+^ permeability. Luckily, except for D-DMAc-11, the change in VEs of ICFBs was particularly minor after coating with Nafion resin, which is attributed to the increasing Nafion resin and the improved hydrophilicity. As expected, by combining the high ion selectivity, an ICFB with a D-DMAc-8 membrane exhibits the best performance (Energy efficiency EE = 75.39%, at the current density of 80 mA cm^−2^), which is close to an ICFB with a Nafion 115 membrane.

**Figure 8:**
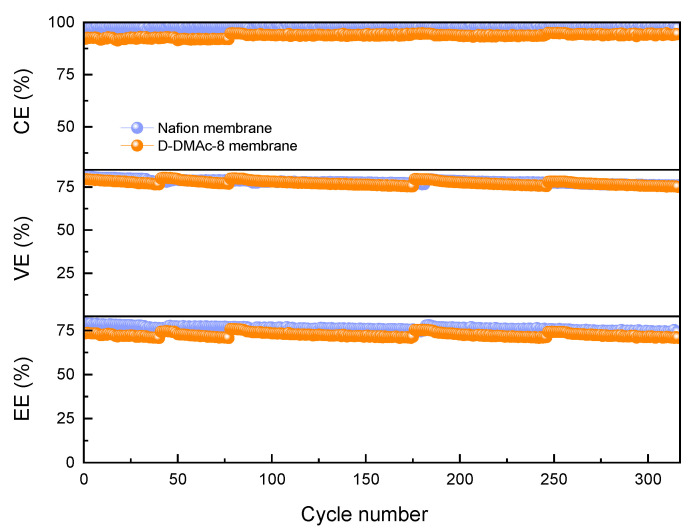
Cycling performance of ICFB assembled with the D-DMAc-8 and Nafion membranes at the current density of 80 mA cm^−2^.

In order to evaluate the stability of ICFBs with D-DMAc-8 and Nafion membranes, long-term operation tests were conducted at the current density of 80 mA cm^−2^ (Figure 8). CEs of ICFBs with D-DMAc-8 membrane are stably maintained at 93% with the increase of the cycle numbers. There is a slight decrease in VEs, which probably arises from the inactivation of redox-active ions. VEs and EEs could be recovered close to the original value via the renewal of the electrolyte. The VEs of ICFBs with a D-DMAc-8 membrane are equivalent to that of a commercial Nafion membrane, and the EEs of ICFB with a D-DMAc-8 membrane are slightly lower than those with Nafion. However, the cost of the D-DMAc-8 membrane is much lower than the Nafion membrane. That is, as the substitute for the Nafion membrane, the D-DMAc-8 membrane is cost-effective and the performance and stability of ICFBs with a D-DMAc-8 membrane are acceptable.

**Figure 9:**
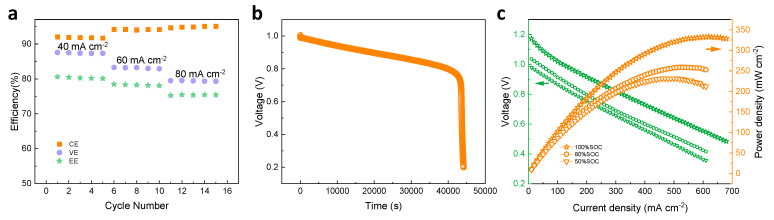
(**a**) The rate performance of an ICFB with D-DMAc-8 membrane at the current density of 40–80 mA cm^−2^; (**b**) OCV curves of ICFBs with D-DMAc-8 membrane; (**c**) polarization curve and power density of an ICFB with D-DMAc-8 membrane at different SOC (100%, 80% and 50%).

Operation current density significantly affects the power density and cost of an ICFB system. To evaluate the rate capability, ICFBs with Daramic and D-DMAc-8 membranes at various current densities were tested. An ICFB with a Daramic membrane cannot operate for the whole rate performance test due to the serious ion cross-over through the large pores at the early stage of the test. An ICFB with a D-DMAc-8 membrane (Figure 9a) operates successfully at the current density of 40–80 mA cm^−2^ and exhibits stable efficiency at a certain set of current density. As the current density increases from 40 mA cm^−2^ to 80 mA cm^−2^, CE increases from 92% to 94% due to the lower ion cross-over during the shorter charge–discharge time, while VE decreases from 87% to 80% due to the rising ohmic polarization.

Besides, the self-discharge test was carried out to evaluate the permeability of redox-active ions of a D-DMAc-8 membrane. The results are shown in Figure 9b. Due to the Nafion resin in pores that effectively inhibits the transfer of redox-active ions, the D-DMAc-8 membrane shows excellent self-discharge performance. The polarization curves and power density of the ICFB with a D-DMAc-8 membrane at different states of charge (SOC) are shown in Figure 9c. An ICFB with a D-DMAc-8 membrane is capable of delivering a limiting current density of 620 mA cm^−2^ and a high peak power density of 333.31 mW cm^−2^ at the SOC of 100%, indicating that it is a great prospect.

### 3.5. The Stability of the Composite Membranes

The harsh operation conditions of ICFBs, such as the high operating temperatures (65 °C), high electrode compression ratio, and high flow rate, put forward higher requirements for the membrane stability. The thermal, mechanical, and chemical stability of the D-DMAc-8 membrane were characterized in detail.

**Figure 10:**
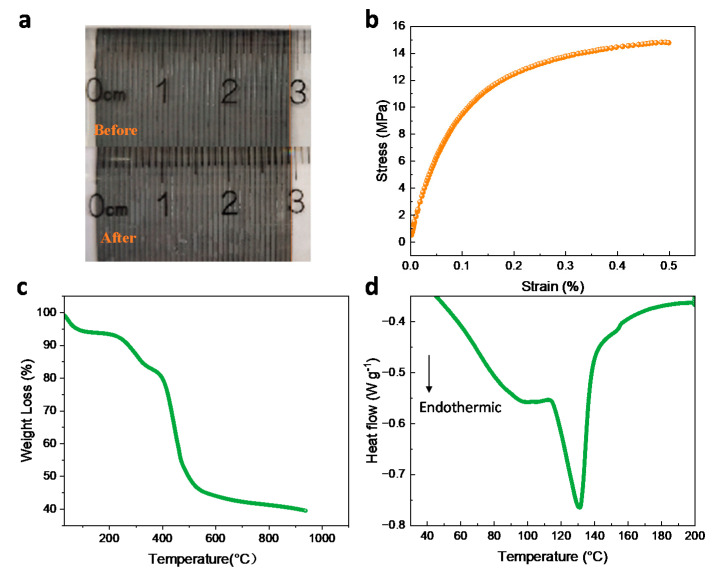
(**a**) Photos showing the swelling of D-DMAc-8 membrane before and after immersing in water for 24 h, (**b**) stress-strain curve, (**c**) TG, and (**d**) DSC analysis of D-DMAc-8 membrane.

As is well known, the widely-used Nafion membrane faces serious swelling in aqueous solution, leading to low ion selectivity and poor mechanical stability. The condition creates the risk of piercing the membrane with a carbon felt electrode with a high compression ratio, especially at high operating temperatures. However, it is worth noting that the size of the D-DMAc-8 membrane hardly changed after soaking (SR = 2%) (Figure 10a), demonstrating satisfactory stability and benefiting from the Daramic membrane as the framework structure.

The mechanical performance of the D-DMAc-8 membrane was tested and shown in Figure 10b. We can see that the stress of the membrane is 14.79 MPa. At the same time, we also tested the puncture resistance (Table S1) and the excellent mechanical properties that could satisfy the requirements of ICFB assembly.

Simultaneously, a thermogravimetric analysis (TG) of the D-DMAc-8 membrane was conducted in nitrogen from 25 °C to 900 °C in Figure 10c. There is little weight loss before 150 °C because the hydrophilic membrane absorbed the water molecules from the air. The mass fell about 460 °C, which corresponds to the decomposition of the polyolefin backbone in the Daramic membrane, while the fall at around 550 °C is attributed to the decomposition of the Nafion polymer backbone.

Figure 10d shows the DSC curve of the D-DMAc-8 membrane during pyrolysis. When the temperature is lower than 200 °C, two endothermic peaks appear. Combined with thermogravimetric analysis, the first endothermic peak is attributed to the loss of water molecules. The crystalline zone of the D-DMAc-8 membrane began to melt at 130.69 °C. The TG and DSC results show that the D-DMAc-8 membrane possesses good thermal stability at the operating temperature range of ICFBs.

To further confirm the chemical stability of the D-DMAc-8 membrane, the membrane was immersed in a Fenton solution at 80 °C for 7 h, and, after measurement, the mass loss of the soaked D-DMAc-8 membrane was less than 6%, suggesting good chemical stability of the membrane.

### 3.6. Cost Calculation

The cost of the D-DMAc-8 membrane and recast Nafion 115 membrane was calculated. Benefiting from the reducing Nafion amount and the cheap host material, the cost of the D-DMAc-8 membrane is less than about 50% of the recast Nafion 115 membrane by Nafion dispersions (Table S2). Zeng et al. analyzed the electrolyte cost of VFBs and ICFBs. Compared with the VFBs with an electrolyte cost of 122 $kWh^−1^, the cost of the active substances in ICFBs is as low as 17 $kWh^−1^ [32]. Combined with the cheap membrane and low-cost electrolyte, ICFBs are economically effective and have a broader application prospect.

## 4. Conclusions

In summary, we successfully fabricated a composite membrane with high stability and low cost specifically for iron–chromium flow batteries (ICFB). Benefiting from the porous framework structure of a Daramic membrane, the D-DMAc-8 membrane exhibited outstanding thermal, mechanical, and chemical stability, thereby conquering the harsh operation situation of ICFBs. Meanwhile, the fill of ion exchange resin ensured high ion conductivity and ion selectivity. Transmission electron microscopy (TEM) and atomic force microscope (AFM) revealed the proton transfer channels and hydrophobic/hydrophilic phase separation structure in the D-DMAc-8 membrane. Consequently, ICFBs assembled with the prepared membranes exhibited excellent battery performance with a CE of 93.29% and a VE of 80.81% at a current density of 80 mA cm^−2^ and ran stably for over 300 cycles, exhibiting high stability. The specifically designed composite membrane was expected to promote the development of ICFBs.

## Figures and Tables

**Table 1:** The solvent composition and property of 5 wt% cast solution for D-H_2_O, D-IPA, and D-DMAc.

Cast Solution	H_2_O Volume Fraction	IPA Volume Fraction	DMAc Volume Fraction	δ	Δδ	Ra	Ra-Ro
5 wt% Nafion (H_2_O)	0.89	0.11	0	45.138	27.338	35.569	27.669
5 wt% Nafion (IPA)	0.085	0.915	0	25.657	7.857	13.281	5.381
5 wt% Nafion (DMAc)	0.085	0.11	0.805	25.013	7.213	11.410	3.510

## Data Availability

The data in this study are available on request from the corresponding author.

## Supplementary Materials

The following supporting information can be downloaded at: https://www.mdpi.com/article/10.3390/polym14112245/s1, Table S1: The mechanical properties of Daramic and D-DMAc-8 membranes; Table S2: The cost of prepared rNafion 115 (126 μm) and D-DMAc-8 membranes per m^2^; Figure S1: The photos of composite membranes by one-step coating way with (a) 5 wt% cast solution, (b) 10 wt% cast solution, (c) 20 wt% cast solution; Figure S2: The photos of composite membranes with (a) one-step coating way, (b) step-by-step coating way; Figure S3: Contact angle of (a) D-DMAc-0, (b) D-DMAc-2, (c) D-DMAc-4, (d) D-DMAc-6, (e) D-DMAc-8, (f) D-DMAc-11 membranes.
