# Supplementary material for: A Composite Membrane with High Stability and Low Cost Specifically for Iron–Chromium Flow Battery
Source: Polymers (Basel). 2022 May 31;14(11):2245. doi: 10.3390/polym14112245 (PMC9182800; doi:10.3390/polym14112245)
Supplement: Supplementary file 1 [file polymers-14-02245-s001.zip › polymers-1732805-supplementary.pdf]

polymers

MDPI

Supplementary Materials: A Composite Membrane with High Stability and Low Cost Specifically for an Iron–Chromium Flow Battery

Lin Qiao, Shumin Liu, Maolin Fang, Mingjun Yang and Xiangkun Ma

1. The calculation of Hansen solubility parameters

1.1. Hansen solubility parameter was defined according to the following Equation (1):

$$\boldsymbol{\delta^2 = \delta_d^2 + \delta_p^2 + \delta_h^2} \quad (1)$$

δ_d, δ_p, and δ_h represent the contribution of the dispersion force, polar force, and H-bonding.

δ, δ_d, δ_p, and δ_h of H_2O, IPA, DMAc, and PE refer to the Hansen Solubility Parameters Handbook.

1.2. Each of the δ_d, δ_p, and δ_h of mixed solvent in cast solution is a linear function of composition and calculated according to the following Equation (2):

$$\boldsymbol{\delta_{blend} \equiv [\varphi_{comp1} \times \sigma_{com1}] + [\varphi_{comp2} \times \sigma_{com2}]} \quad (2)$$

φ represents the volume fraction for each component.

1.3. Solubility parameter "distance" (Ra) between the mixed solvent and PE was defined according to the following Equation (3):

$$\boldsymbol{(Ra)^2 = 4(\delta_{D1} - \delta_{D1})^2 + (\delta_{P2} - \delta_{P1})^2 + (\delta_{H2} - \delta_{H1})^2} \quad (3)$$

The experimentally-determined solubility sphere radius (Ro) of PE is 7.9—the reference is from Hansen Solubility Parameters Handbook.

Table S1. The mechanical properties of Daramic and D-DMAc-8 membranes.

Thickness (μm)	Code	Mechanical Property			Puncture Strength (N)
		Elastic Modulus (MPa)	Elongation at Break (%)	Tensile Strength (MPa)	
200 ± 5	Daramic membrane	119.57	49.22	15.08	
200 ± 5	D-DMAc-8 membrane	156.34	50	14.83	4.355

Table S2. The cost of prepared Nafion 115 (126 μm) and D-DMAc-8 membranes per m^2.

Code	Component	Unit Price ($)	Consumption	Cost ($)
Nafion 115 membrane	Nafion dispersions (D-2020)	2.916 mL-1	630 mL	1837.08
Total				1852.776
	DMAc	0.004993 mL-1	937.5 mL	4.68

D-DMAc-8 membrane	Nafion dispersions (D-2020)	2.916 mL-1	312.5 mL	911.25
	Daramic membrane	31.525 m-2	1 m2	31.525
Total				947.45

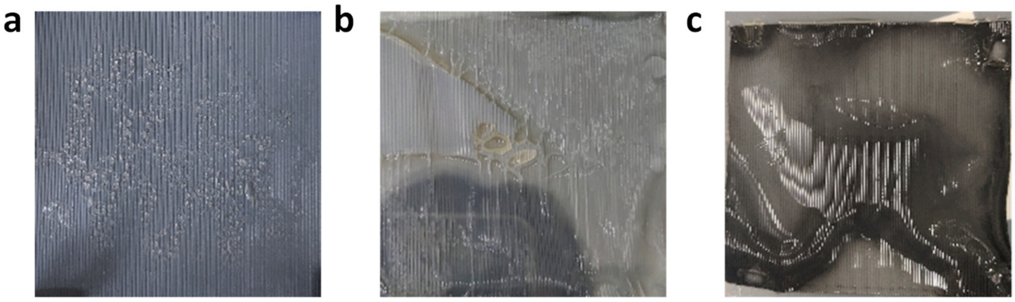

Figure S1. The photos of composite membranes by one-step coating way with (**a**) 5 wt% cast solution, (**b**) 10 wt% cast solution, and (**c**) 20 wt% cast solution.

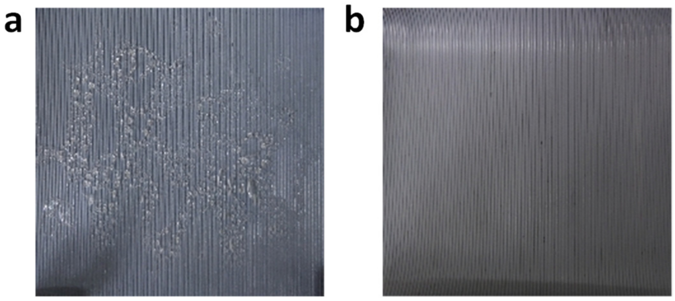

Figure S2. The photos of composite membranes with (**a**) one-step coating way and (**b**) step-by-step coating way.

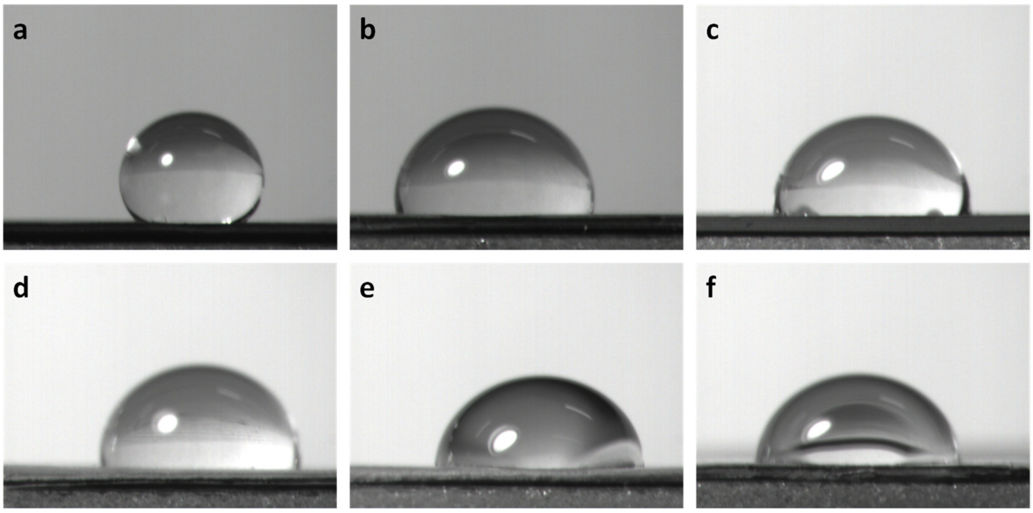

Figure S3. Contact angle of (**a**) D-DMAc-0, (**b**) D-DMAc-2, (**c**) D-DMAc-4, (**d**) D-DMAc-6, (**e**) D-DMAc-8, (**f**) D-DMAc-11 membranes.
